# The retinal foveal avascular zone as a systemic biomarker to evaluate inflammatory bowel disease control

**DOI:** 10.1186/s40942-019-0168-9

**Published:** 2019-08-06

**Authors:** Luis Filpe Nakayama, Vinicius Campos Bergamo, Marina Lourenco Conti, Nikoly Tigani Fares, Livia Almeida Costa, Orlando Ambrogini, Nilva Simeren Bueno de Moraes

**Affiliations:** 1 Department of Ophthalmology, Universidade Federal de São Paulo—EPM, Rua Guarujá, 326 ap 54, São Paulo, SP 04052-110 Brazil; 2 Department of Medicine, Universidade Federal de São Paulo—EPM, São Paulo, SP Brazil

**Keywords:** Retina, Inflammatory bowel disease, Optical coherence tomography, Optical coherence tomography angiography, Foveal avascular zone

## Abstract

**Background:**

Inflammatory bowel disease (IBD) is a systemic inflammatory disease and is classified as Crohn’s disease (CD) or ulcerative colitis (UC) depending on the extent of gastrointestinal tract involvement. IBD can be associated with extraintestinal findings, such as fever, weight loss, arthralgia, and mucocutaneous lesions, as well as hepatic, renal and ophthalmological involvement. Clinical parameters and colonoscopy are used to establish the criteria for controlled or non-controlled disease and subsequent definition of treatment. Our objective in the present study was to compare the area of the foveal avascular zone (FAZ) in patients with a diagnosis of IBD during remission and active disease.

**Methods:**

144 eyes of 72 patients with IBD were evaluated via a complete ophthalmological exam. Fundus photography and optical coherence tomography/angiography (OCT/OCTA) were performed with a Topcon Triton. The macula and posterior pole were evaluated by binocular indirect ophthalmoscopy and fundus biomicroscopy. The area of the FAZ was determined via manual delimitation of superficial retinal vascular layers from OCTA with image6.net software. To establish disease activity, we considered the Mayo Score, fecal calprotectin levels, colonoscopy results and clinical parameters. All retinal parameters were evaluated in a blinded manner. Means were compared between groups using the Mann–Whitney test.

**Results:**

The participants had a mean age of 42.26 years and included 28 males (38.88%) and 44 females (61.11%). Among the participants, 37 had a diagnosis of CD (51.38%), and 35 had a diagnosis of UC (48.61%). Twenty-five patients (34.72%) had active disease, and 47 (65.27%) were in remission. The area of the FAZ did not differ significantly between the CD and UC groups (*p* = 0.91 for the right eye and *p* = 0.76 for the left eye) but did differ significantly between the remission and active disease groups (*p* = 0.01 for the right eye and *p* = 0.02 for the left eye).

**Discussion:**

Our study is the first to evaluate the area of the FAZ in patients with IBD via swept-source OCTA. The area of the FAZ did not differ significantly in either eye between the CD and UC groups. However, patients classified as having active disease according to clinical parameters and colonoscopy presented a significant decrease in the area of the FAZ compared with patients in remission. The area of the FAZ is an ophthalmological parameter that can be obtained non-invasively and is increased in ischemic diseases such as diabetic retinopathy. The FAZ may decrease due to vascular engorgement or increased systemic inflammation. This parameter can be used to help determine whether a patient is in remission or active IBD, thus potentially reducing the need for invasive exams during disease follow-up.

## Introduction

Crohn’s disease (CD) and ulcerative colitis (UC) are systemic inflammatory diseases but mainly feature gastrointestinal involvement. Both CD and UC are forms of inflammatory bowel disease (IBD) and can be classified according to gastrointestinal tract and histological involvement. CD and UC are immune mediated and affect 1.5 million Americans and 2.2 million Europeans, with increasing incidence in recent decades. Reliable Brazilian prevalence data are lacking [1, 2]. UC is characterized by distal to proximal involvement restricted to superficial intestinal mucous layers. CD features more extensive involvement in which all intestinal layers are compromised [3].

The exact pathophysiology of IBD is not completely known but involves the interaction of genetic, ambient, psychological, microbiological and immunological factors [1]. Sedentarism, smoking, use of non-steroidal anti-inflammatory drugs, menopausal hormone therapy, living in urban areas, precarious hygiene conditions, living in northern latitudes, and certain ethnic backgrounds, such as Ashkenazi Jewish populations, are risk factors for IBD [1].

Extraintestinal symptoms are the first manifestation of IBD in 10–20% of cases [4]. The pathophysiology of these symptoms involves a combination of allergic and immune reactions, with immune complex deposits in extraintestinal sites. Hepatobiliary, renal, joint, mucocutaneous, general and ophthalmological involvement may occur [5].

Ophthalmological involvement is present in approximately 3.5%–12% of IBD patients [4, 6–8] and is usually more common in the initial years of the disease. Ophthalmological involvement is not directly correlated with disease activity [9]. Ophthalmological symptoms may include palpebral edema, blepharitis, proptosis, conjunctivitis, scleritis, episcleritis, keratopathy, cataract, optic neuritis, optic, neuropathy, iridociclitis, retinal pigment epithelial atrophy, macular edema, serous retinal detachment, vascular tortuosity and retinal hemorrhage [5, 9, 10]. Ophthalmological posterior segment findings are described in 26% of patients with IBD and are very relevant because they can lead to irreversible blindness in cases of optic neuritis, optic neuropathy and retinal atrophy [5, 9].

Our study objectives were to compare the area of the foveal avascular zone (FAZ) in patients with an IBD diagnosis and to attempt to correlate the area of the FAZ with disease activity.

## Methods

This was a cross-sectional study of 144 eyes of 72 patients from the Gastroenterology sector of Paulista Medical School/Federal University of São Paulo (UNIFESP) with a diagnosis of IBD evaluated at the Ophthalmology department. This study was conducted according to the principles of the Declaration of Helsinki under UNIFESP Ethics Committee approval (Number 1453/2016). All patients were informed about the ophthalmological exams and procedures and signed a participation consent form.

All patients underwent a complete ophthalmological examination during the same period of the day by the same ophthalmologist. Posterior segment exams were performed with a Triton swept-source optic coherence tomography (OCT) device (Topcon^®^, Tokyo, Japan). All patients underwent color fundus photography, swept-source macular OCT, and optical coherence tomography angiography (OCTA).

**Fig. 1:**
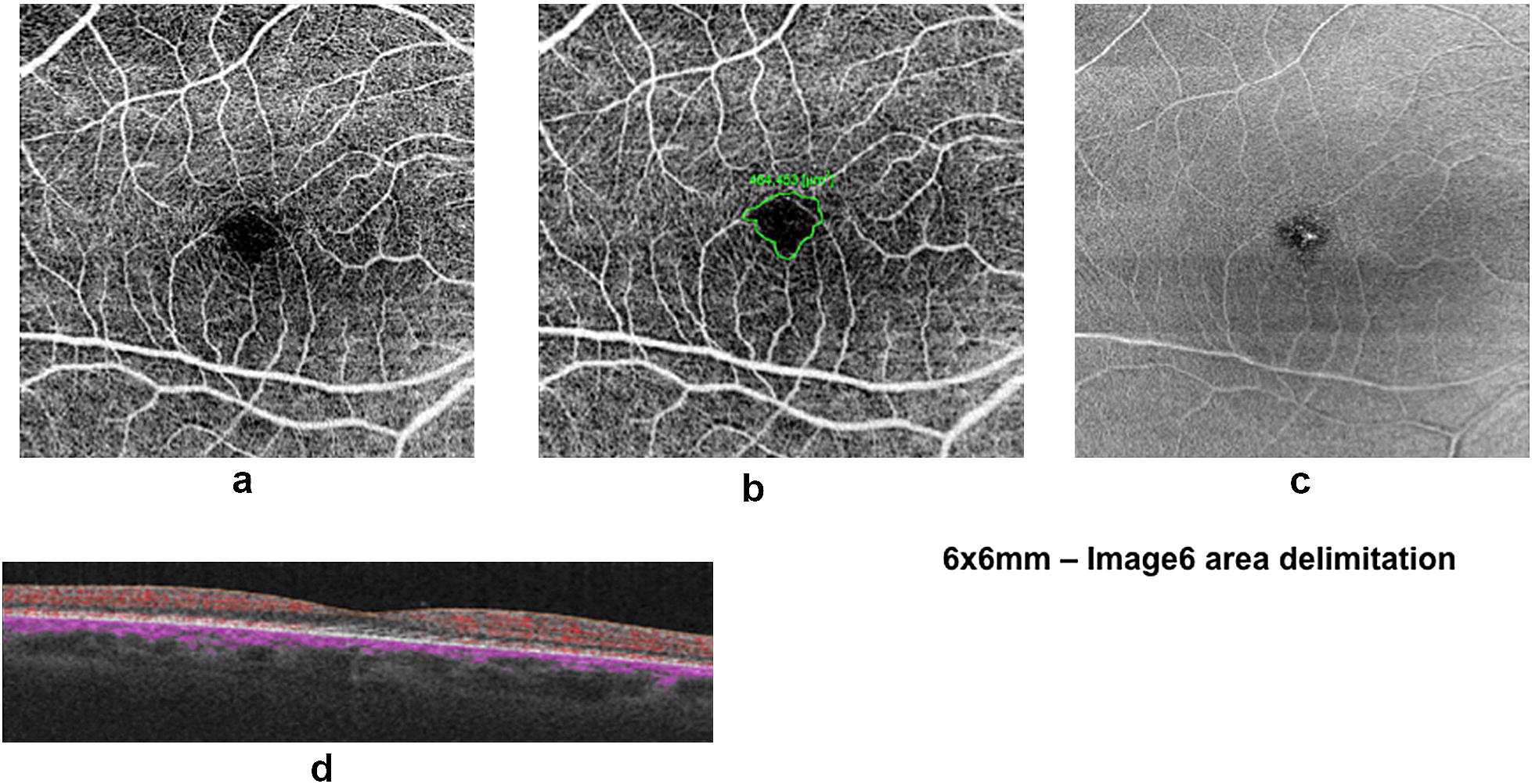
Foveal Avascular Zone in 6X6 OCTA. **a** Superificial Vascular layer. **b** Foveal Avascular Zone. **c** En face superficial slab. **d** Corresponding OCT

The FAZ area was manually determined by image6.net software analysis of a 6 × 6 mm superficial OCTA layer exam by a single retina specialist. To exclude artifacts, we evaluated OCT scan quality and en-face slabs (Fig. 1).

Patients with recent ophthalmological surgery, low-quality exams, refractive errors greater than + 3 or − 3 spherical and retinopathies such as diabetic retinopathy were excluded.

To establish disease activity, gastroenterology specialists considered the Mayo Score for Ulcerative Colitis (higher scores indicate more severe IBD) [11], calprotectin levels (normal inferior to 50 mcg/g) [12], colonoscopy findings and clinical parameters and classified the disease as in remission or active.

The area of the FAZ was compared between the CD and UC groups and between the active disease and remission groups. All retinal parameters were evaluated in a blinded manner. Means were compared between groups using Mann–Whitney test.

## Results

In total, 72 patients were enrolled in the study. The mean age of the participants was 42.26 years (maximum of 68 years and 7 months and minimum of 18 years and 1 month). Among the participants, 28 were male (38.88%), and 44 were female (61.11%). CD was diagnosed in 37 patients (51.38%), and UC was diagnosed in 35 patients (48.61%). The mean time since disease diagnosis was 10.78 years (1 year minimum and 36 years maximum). Twenty-five patients (34.72%) had active disease, and 47 (65.27%) were in remission (Table 1).

**Table 1:** Patients demographics and IBD characteristics

Age	Mean: 42.08 years	Min: 18 years	Max: 68 years
Gender	Males: 28	Females: 44	
Diagnosis	CD: 37	UC: 35	
Disease time	Mean: 10.78 yrs	Min: 1 year	Max: 36 years
Disease control	Active: 21	Remission: 51	

The mean area of the FAZ in the UC group was 0.31 mm^2^ (standard deviation of 0.14 mm^2^) in the right eye and 0.31 mm^2^ (standard deviation of 0.15 mm^2^) in the left eye. In the CD group, the mean area of the FAZ was 0.31 mm^2^ (standard deviation of 0.13 mm^2^) in the right eye and 0.32 mm^2^ (standard deviation of 0.15 mm^2^) in the left eye. The difference in the mean area of the FAZ between the UC and CD groups was not significant, with a p of 0.91 for the right eye and 0.76 for the left eye.

In the group with active disease, the mean FAZ area was 0.25 mm^2^ (standard deviation of 0.11 mm^2^) in the right eye and 0.25 mm^2^ (standard deviation of 0.13 mm^2^) in the left eye. In the remission group, the mean FAZ area was 0.34 mm^2^ (standard deviation of 0.13 mm^2^) in the right eye and 0.34 mm^2^ (standard deviation of 0.15 mm^2^) in the left eye.

The difference in the mean FAZ between the active disease and remission groups was significant for both the right (*p* = 0.01) and left eyes (*p* = 0.02) (Table 2).

**Table 2:** Fovel Avascular zone

	Righ eye	Left eye
Ulcerative colitis	0.31 mm^2^ (sd 0.14 mm^2^)	0.31 mm^2^ (sd 0.15 mm^2^)
Crohn disease	0.31 mm^2^ (sd 0.13 mm^2^)	0.32 mm^2^ (sd 0.15 mm^2^)
	p 0.91	p 0.76
Active IBD	0.34 mm^2^ (sd 0.13 mm^2^)	0.34 mm^2^ (sd 0.15 mm^2^)
Remission IBD	0.25 mm^2^ (sd 0.11 mm^2^)	0.25 mm^2^ (sd 0.13 mm^2^)
	p 0.01	p 0.02

None of the study participants had a history of ocular surgery or pathology.

## Discussion

IBD is a systemic inflammatory disease with gastrointestinal symptoms and extraintestinal manifestations. IBD may present ophthalmological involvement in 3–12% of cases, with episcleritis as the most prevalent finding and vascular tortuosity as the most prevalent fundoscopic finding [4, 6–8, 10]. IBD may be classified as active or in remission according to clinical gastrointestinal symptoms and colonoscopy parameters. Our study is the first to evaluate the FAZ area in patients with IBD in order to compare UC patients with CD patients or patients in remission with those with active disease.

OCT is a non-invasive, rapid exam that permits the analysis of different retinal slabs through optic interferometry [13]. OCTA uses the same technology to evaluate the vascular plexus in different layers and to perform en-face analysis. The area of the FAZ may be determined from superficial/deep retinal vascular OCTA en-face images and is correlated with retinal microcirculation parameters. The area of the FAZ is increased in systemic ischemic vascular diseases such as diabetic retinopathy [14].

No significant difference in FAZ area was observed between the UC and CD groups. In addition, there was no significant difference in mean values between the right and left eyes. By contrast, when patients with active disease or in remission were compared, the mean FAZ area was significantly smaller in both the right and left eyes of patients with active disease. A smaller FAZ may be correlated with systemic microvascular engorgement and increased systemic inflammation in active IBD, as an indirect sign of disease activity.

More studies are necessary to establish the exact cause-effect relationship, including a prospective analysis of these ophthalmological parameters in patients during active IBD and after disease remission.

## Conclusion

The area of the FAZ is as an ophthalmological parameter that may be obtained non-invasively via OCTA. Evaluation of the FAZ could help determine if a patient is in remission or has active IBD disease, thus potentially reducing the need for invasive exams during systemic follow-up.

## Data Availability

The datasets generated during the current study that were used to calculate the primary outcome parameters are available upon reasonable request from the corresponding author Nakayama, LF.
